# Prediction of Static Modulus and Compressive Strength of Concrete from Dynamic Modulus Associated with Wave Velocity and Resonance Frequency Using Machine Learning Techniques

**DOI:** 10.3390/ma13132886

**Published:** 2020-06-27

**Authors:** Jong Yil Park, Sung-Han Sim, Young Geun Yoon, Tae Keun Oh

**Affiliations:** 1 Department of Safety Engineering, Seoul National University of Science and Technology, Seoul 01811, Korea; jip111@seoultech.ac.kr; 2 School of Civil, Architectural Engineering and Landscape Architecture, Sungkyunkwan University, Suwon 16419, Korea; ssim@skku.edu; 3 Department of Safety Engineering, Incheon National University, Incheon 22012, Korea; 4 Research Institute for Engineering and Technology, Incheon National University, Incheon 22012, Korea

**Keywords:** concrete, static elastic modulus, dynamic elastic modulus, compressive strength, machine learning, P-wave, S-wave, resonance frequency test, nondestructive method

## Abstract

The static elastic modulus (*Ec*) and compressive strength (*fc*) are critical properties of concrete. When determining *Ec* and *fc*, concrete cores are collected and subjected to destructive tests. However, destructive tests require certain test permissions and large sample sizes. Hence, it is preferable to predict *Ec* using the dynamic elastic modulus (*Ed*), through nondestructive evaluations. A resonance frequency test performed according to ASTM C215-14 and a pressure wave (P-wave) measurement conducted according to ASTM C597M-16 are typically used to determine *Ed*. Recently, developments in transducers have enabled the measurement of a shear wave (S-wave) velocities in concrete. Although various equations have been proposed for estimating *Ec* and *fc* from *Ed*, their results deviate from experimental values. Thus, it is necessary to obtain a reliable *Ed* value for accurately predicting *Ec* and *fc*. In this study, *Ed* values were experimentally obtained from P-wave and S-wave velocities in the longitudinal and transverse modes; *Ec* and *fc* values were predicted using these *Ed* values through four machine learning (ML) methods: support vector machine, artificial neural networks, ensembles, and linear regression. Using ML, the prediction accuracy of *Ec* and *fc* was improved by 2.5–5% and 7–9%, respectively, compared with the accuracy obtained using classical or normal-regression equations. By combining ML methods, the accuracy of the predicted *Ec* and *fc* was improved by 0.5% and 1.5%, respectively, compared with the optimal single variable results.

## 1. Introduction

During the design, construction, and maintenance of concrete structures, static elastic modulus (*Ec*) and compressive strength (*fc*) are critical properties for analyzing structural stability parameters such as member force, stress, deflection, and displacement [1,2]. In addition, these properties are indicators of concrete deterioration. Therefore, *Ec* and *fc* have been used to evaluate the conditions of structures such as pavements and bridge decks. The specimen cores of existing concrete structures are typically extracted and tested to determine *Ec* and *fc,* according to the recommended ASTM C469/C469M-14 guidelines. *Ec* is typically predicted from *fc*; however, this yields a relatively large error [3]. The standard testing methods for determining *Ec* and *fc* cannot be used to evaluate the entire area, owing to the need for test permissions and several specimens. Furthermore, the values of *Ec* and *fc* vary at different locations in the concrete structure, and these differences may increase with aging. Therefore, it is necessary to determine *Ec* and *fc* using a nondestructive evaluation (NDE) method that can be applied at several locations in a structure without damaging the structure.

The elastic modulus measured using the NDE method is generally called the dynamic elastic modulus (*Ed*). Ultrasonic pulse velocity (UPV) measurements based on the ASTM C597/ASTM C597M-16 guidelines and resonance frequency tests conducted in accordance with the ASTM C215-14 specifications are used to determine *Ed* [4,5]. As for ASTM C215, Subramaniam et al. have improved the standard equation using the second resonance frequency as well as the first. Although it has the advantage of finding the dynamic Poisson ratio, its accuracy is insignificant [6]. The *Ed* determined with the NDE method is generally higher than the *Ec* obtained by following the ASTM C469/C469M-14 recommendations [7]. The *Ed* determined with the P-wave measurement was the largest, and the *Ed* determined with the resonant frequency test was located in the middle compared to *Ec* [8,9]. The *Ed* value with the S-wave measurement was more correlated with *Ec* and *fc* than the *Ed* using the P-wave measurement and resonant frequency [10]. However, the S-wave is difficult to measure, and the dispersion of the measured values may be large at a low age. Additionally, it is more difficult to predict the properties of early aged and low-strength concrete (which include moisture and voids) and high-strength concrete. It has been known that the static modulus (secant modulus of elasticity) determined by the destructive test according to ASTM C469 and the dynamic modulus determined by non-destructive tests such as ASTM C597 and ASTM C215 have a nonlinear proportional relationship, and the static modulus is 10–20% less than the dynamic modulus [11]. Since the dynamic modulus is determined by the slope at a very low stress range, it is less statistically stable than the elastic modulus, but it has the advantage of being simple to test without the damaging of the specimen [12]. Therefore, it is essential to develop a method that can be used to more accurately predict *Ec* from *Ed* because it is difficult to measure all the specimens with the destructive test.

Various equations have been proposed for predicting *Ec* based on *Ed*. For example, Lydon and Balendran, BS 8110 Part 2, and Popovics proposed empirical equations for predicting *Ec* from *Ed,* using the NDE method [11,12,13]. Among previously proposed equations, the BS 8110 Part 2 equation yields similar values to the *Ed* value measured using the UPV through P-waves, and the Popovics equation provides results that are similar to *Ed* obtained through resonance frequency tests using longitudinal and transverse modes [10]. In addition, the Lydon and Balendran results are lower than those obtained using other equations [9]. Most of the previous studies attempted to relate Ec values with Ed values using statistical regression analysis such as linear or nonlinear regression [8]. However, these equations may lead to a significant error in predicting *Ec* if the Ed value obtained from another test method is used. As described above, because the deviation and the degree of change in the *Ed* value differs significantly for each NDE method, it is difficult to apply these equations for the accurate prediction of *Ec*.

Additionally, correlation equations have been proposed by CEB-FIP, the ACI 318 Committee, and the ACI 363 Committee as methods for predicting *fc* using *Ed* [14,15,16]. However, when predicting *fc* using *Ed*, the prediction error may increase in a step-by-step relational expression because the procedures for predicting *Ec* in *Ed* and *fc* in *Ec* are employed. Therefore, it is necessary to apply a method that can solve the problems of the existing prediction of linear regression and predict *Ec* and fc more accurately by utilizing various *Ed* that can be obtained through experiments. Recently, Chavhan and Vyawahare [17] proposed a study to predict fc from *Ed* with P-wave velocity tests but did not obtain a good relationship, even with only 22 specimens. Thus, for the relationship between *Ec* or fc and *Ed,* the classical or regression equations could not give an accurate estimation for *Ec* from *Ed* because the *Ed* value differs according to the test method [18]. Additionally, there exists considerable nonlinearity between *Ec* or *fc* and *Ed* [19].

In this regard, the machine learning (ML) technology has been applied and improved in many areas of concrete, but it has been used to predict *Ec* and *fc* limitedly with various components [20], to establish a correlation between *Ec* and fc [21], and to estimate fc from ultrasonic velocity and rebound hardness [22]. Furthermore, the estimation of *Ec* or fc from *Ed* has been little studied due to the difficulties in testing enough specimens. Thus, these studies have focused on obtaining more accurate predictions of concrete strength and conditions using (ML) algorithms such as support vector machine (SVM), ensemble, and artificial neural networks (ANNs) [21,22,23,24].

In most studies, ML methods contributed to predicting fc with higher accuracy than conventional linear regression methods. In addition, it was used to predict the characteristics of concrete using various variables as follows. Erdal et al. used an ANN and ensemble for comparing the accuracy of strength prediction for high-performance concrete, and the method of applying the ensemble was found to be slightly better [25]. Yuvaraj et al. confirmed the applicability of several variables to the SVM model by predicting the fracture characteristics of concrete beams according to the ratio of concrete mixtures from the SVM model [26]. Yan and Shi used four theoretical equations, linear regression, and ANN and SVM models to confirm the accuracy of *Ec* prediction with *fc*, and the ANN and SVM methods were found to be the best methods [27]. Cihan employed SVM, ANN, and ensemble to predict the compressive strength and slump value of concrete, and reported that ANN and ensemble were better than SVM [28]. Young et al. employed SVM, ANN, and linear regression (LR) to estimate 28-day intensity from 10,000 data with varying mixing ratios and reported that ANN and SVM were more accurate than LR [29]. Among the many ML methods, the SVM, ANN, ensemble, and LR methods contributed to the prediction of concrete quality using various variables. However, previous studies only considered cases such as the prediction of strength and the modulus of elasticity using different combinations of materials, ultrasonic pulse velocity, and *fc*. Very few studies have attempted to predict *Ec* and *fc* using two or more *Eds* and ML.

In this study, various combinations were used to overcome the differences in the characteristics of the eigen-data of *Eds* through the ML methods and to confirm the possibility of predicting actual *Ec* and *fc* values more accurately than conventional linear equations. Various *Ed* values were obtained with UPV measurements and resonance frequency tests. Additionally, by determining the S-wave velocity (*Vs*), according to the recommended guidelines of ASTM C215-14 and ASTM C597M-16 and by determining *Ed* using *Vs*, the differences with respect to existing *Ed* values were verified and then utilized to improve the accuracy of the prediction. Four ML methods—SVM, ANN, ensemble, and LR—were trained on four *Ed* datasets, and a five-fold validation was performed to prevent the overfitting of the prediction results. Subsequently, the predicted and measured values for *Ec, fc*, and Ed were compared using the mean squared error (MSE) and mean absolute percentage error (MAPE). Thus, this study aimed to confirm how much accuracy can be improved compared to that obtained with the classical and regression equation by predicting *Ec* and *fc* directly from the Ed value, which is a representative property value in the non-destructive testing method, associated with ML. An attempt was made to predict exactly how much and how the *Ec* and *fc* values will be affected not only by the four ML methods that can predict Ed but also by a combination thereof.

## 2. Materials and Methods

### 2.1. Materials and Preparation of Specimens

The concrete specimens were composed of Type I Portland cement, river sand, crushed granite with a size of up to 25 mm, and supplementary cementitious materials (SCMs), i.e., fly ash and slag cement. The concrete of this study replaces about 50% of the cement with fly ash (FA) and Granulated Blast Furnace Slag (GBFS), and is composed of a mixture that causes the development of the initial strength to be slow at a low age but the long-term strength to be high. It is mainly used in mass concrete that generates less heat of hydration and requires long-term strength. Two water/binder (W/B) ratios of 0.45 and 0.35 were used, which were expected to achieve 28-day target compressive strength values of 20 MPa and 40 MPa, respectively. Two concrete mixture groups—Mix 1 and Mix 2—were prepared; the proportions of these mixture groups are presented in Table 1. Two hundred and ninety-five concrete specimens were cast using 150 mm × 300 mm plastic molds, in accordance with the ASTM C31/C31M-12 specifications [30]. The specimens were removed from the molds after 24 h and then cured in water. *Ed, Ec*, and *fc* tests were performed at the different curing ages of 4, 7, 14, and 28 d. The 28-day average compressive strengths for Mixes 1 and 2 were 19.19 MPa and 43.94 MPa, respectively.

### 2.2. Static Tests for Elastic Modulus and Compressive Strength

**Figure 1:**
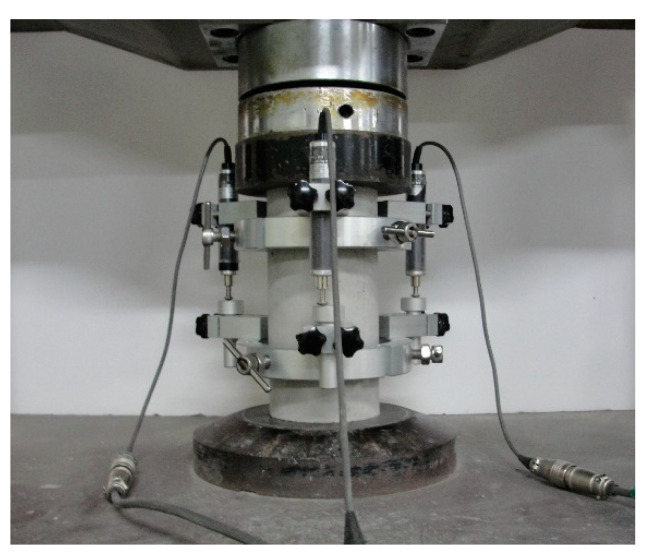
Experimental setup for determining static elastic modulus and compressive strength of concrete specimens.

As a pretest preparation procedure, both ends of each specimen were polished and kept perpendicular to the measurement by removing protrusions on the specimen surface. The *Ec* and *fc* values of each specimen were determined using a universal testing machine (UTM, Instron, Norwood MA, USA) with a capacity of 1000 kN, according to the ASTM C469/C469M-14 and ASTM C39/C39M-14a specifications (Figure 1) [7,31]. The UTM was operated at a loading speed of approximately 0.28 MPa/s.

### 2.3. P- and S-Wave Measurements for Calculating Dynamic Elastic Modulus

**Figure 2:**
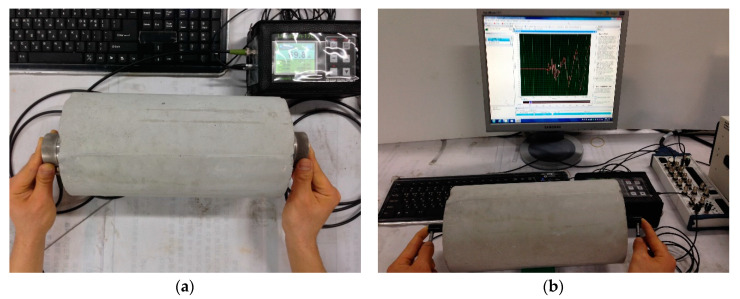
Methods for measuring (**a**) ultrasonic pulse velocity using a P-wave transducer (MK 954) and (**b**) ultrasonic shear-wave velocity using an S-wave transducer (ACS T1802).

The P-wave velocity (*Vp*) was determined according to the ASTM C597M-16/C597M-16 recommendations, using a pair of P-wave transducers (MK 954, MKC Korea, Seoul, Korea) connected to a pulser-receiver (Ultracon 170, MKC Korea, Seoul, Korea) [4]. Figure 2a depicts the method of determining *Vp* using a receiving transducer. A longitudinal pulse of 52 kHz was transmitted through the specimens’ interior, and the *Vp* values of the concrete specimens were determined. The measured signal was digitized to a sampling frequency of 10 MHz using an oscilloscope (NI-PXIe 6366, National Instruments, Austin, TX, USA). The *Ed* based on *Vp* is expressed as Equation (1):

(1) $$Ed.Vp=\frac{\left(1+\nu \right)\left(1-2\nu \right)}{\left(1-\nu \right)}\rho V_{p}^{2}$$

where *Ed.Vp* is the *Ed* of the specimen for the experimentally measured *Vp*, and *ρ* is the mass density of the concrete; it is equal to m/2πrL. *m*, *r*, and *L* are the mass, radius, and length of the specimens, respectively. A Poisson’s ratio (ν) of 0.2 was used, which is the typical value for ordinary concrete.

The S-wave velocity *(Vs)* was determined using the measurement procedure for *Vp*. The measurement of *Vs* was similar to that of *Vp*, except that a pair of S-wave transducers (ACS T1802, ACS Group, Saarbrücken, Germany) were used. The signal driven using the pulsar was converted to a pulse in the transverse mode by the transducer and passed through the specimen. The signal measured using the receiving transducer was digitized using the oscilloscope. Figure 2b presents the method of measuring *Vs* and the ultrasonic signal measured using the S-wave receiver. The arrival of a stress wave through the specimen was determined based on the signals measured using a modified threshold method [32]. In this method, the approximate arrival time was first obtained using a threshold method [33]. Thereafter, the exact arrival time required to fit the line to a single datum was calculated. The S-wave travel time was defined at the intersection between the calculated zero signal level and the fitting line. Finally, *Vs* was calculated by dividing the length of the specimen (*L*) by the travel time (*t*) (*Vs* = *L*/*t*). In this study, the *Ed* based on *Vs* is expressed as Equation (2):

(2) $$Ed.Vs=2\left(1+\nu \right)\rho V_{s}^{2}$$

where *Ed*.*Vs* is the *Ed* of the concrete specimen for the measured *Vs*, and ν is the Poisson’s ratio.

### 2.4. Resonance Frequency Tests for Calculating Dynamic Elastic Modulus

**Figure 3:**
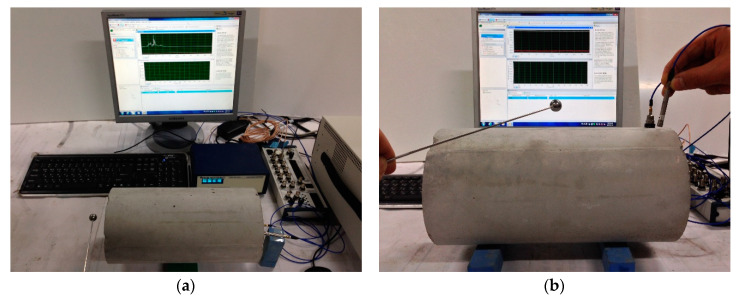
Resonance frequency test for (**a**) longitudinal mode and (**b**) transverse mode.

The fundamental longitudinal and transverse resonance frequencies of the concrete specimens were determined to calculate the *Ed* value according to the ASTM C215-14 guidelines (Figure 3) [5]. A steel ball hammer with a diameter of 10 mm was used to generate stress waves in the concrete specimens through impact. The use of the steel ball hammer was effective in generating significantly low to 20 kHz frequency signals during the resonance frequency tests. The dynamic responses of the concrete specimens were measured using an accelerometer (PCB 353B16, PCB, Erie County, NY, USA) with a resonance frequency of approximately 70 kHz and an error of 5%, within the range of 1 to 10 kHz. The signals measured using the accelerometer were stabilized using a signal conditioner (PCB 482C16, PCB, Erie County, NY, USA) and digitized at a sampling frequency of 1 MHz using an oscilloscope (NI-PXIe 6366). The time signal was transformed into the frequency domain through a fast Fourier transform algorithm. The resonance frequency had the largest amplitude in the amplitude spectrum, and the frequency corresponding to the largest amplitude was considered as the fundamental resonance frequency in the longitudinal and transverse modes. The *Ed* based on this resonance frequency is defined using Equations (3) and (4):

(3) $$Ed.LT=\alpha_{LT}mf_{LT}^{2}\left(Pa\right)$$

(4) $$Ed.TR=\alpha_{TR}mf_{TR}^{2}\left(Pa\right)$$

where *Ed.LT* and *Ed.TR* are calculated using $f_{LT}$ and $f_{TR}$, respectively, as the fundamental resonance frequencies. $\alpha_{LT}$ has a constant value depending on the dimensions of the specimen (5.093 ($L/d^{2}$)), where *L* is the length and d is the diameter. $\alpha_{TR}$ has a constant value, and its value depends on the +Poisson’s ratio and specimen dimensions (1.6067 ($L^{3}T/d^{4}$)), where *T* is the correction factor of the Poisson’s ratio according to ASTM C215-14 [5], and m is the mass of the specimen in kg.

## 3. ML Techniques

### 3.1. SVM

**Figure 4:**
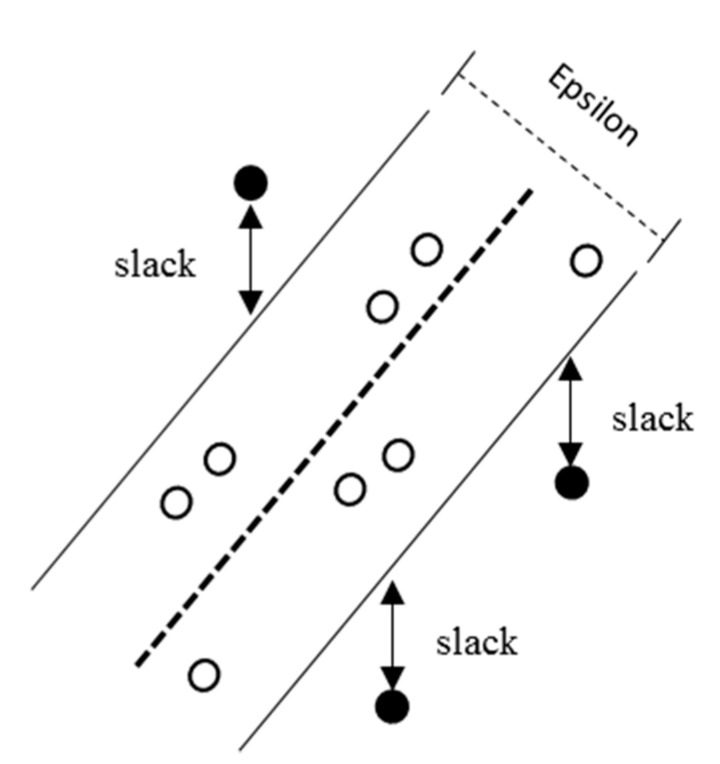
Procedure of support vector regression from an input sample.

The SVM analytical method, proposed by Vapnik in 1992, is a widely used ML tool for classification and regression [34]. SVM regression is considered a nonparametric technique because it is based on kernel functions, where a linear epsilon neglected during SVM (ε-SVM) regression is implemented; it is also known as L1 loss. In ε-SVM regression, the training data set contains values of both predictor variables and observed response variables. For each training point x, the SVM deviates from yn (the response variable value) by a value not higher than ε, as shown in Figure 4. Simultaneously, the objective is to determine the maximum flattened function f(x), as expressed in Equation (5):

(5) $$f\left(x\right)=x^{\prime }\beta +b$$

where β is the linear predictor coefficient, and *b* is the bias. In this study, the SVM regression model was implemented in the MATLAB R2019b (MathWorks, Natick, MA, USA) environment using “fitrsvm”, which employs linear learning.

### 3.2. ANN

**Figure 5:**
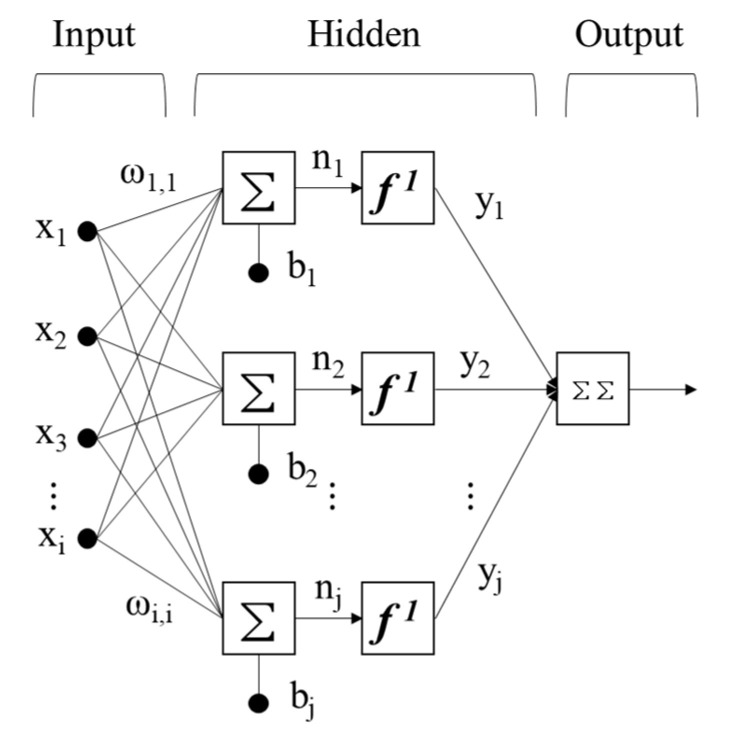
Procedure of an artificial neural network from an input sample.

An ANN is an information processing system based on the structure and function of biological neural networks. ANNs utilize connected artificial neurons and learn input parameters through these neurons to explain their unique behaviors, thereby performing nonlinear modeling. Multilayer perceptron (MLP) is the most commonly used ANN architecture; it consists of input, hidden, and output layers as shown in Figure 5 [35]. As shown in Equation (6), all the neuron connections in every layer of an ANN include weights and biases. The neuron values of previous layers are adjusted by varying weights and compensated through bias.

(6) $$y_{i}=f\left(net\right)=f\left(\overset{n}{\underset{i=1}{\sum }}\omega_{i}x_{i}+b_{j}\right)$$

where *x* is the input value (*i* = 1, ..., *n*), w is the weight vector between neurons, *b* is the bias of the neuron, f is the activation function, and *y* is the output value. MLP is generally trained using an inverse propagation algorithm wherein interconnected weights of the network are repeatedly modified to minimize errors defined as the root mean square errors (RMSEs) [36]. Previous studies have reported that the Levenberg–Marquardt algorithm is suitable because it yields consistent results for the majority of ANNs [37].

### 3.3. Ensemble

**Figure 6:**
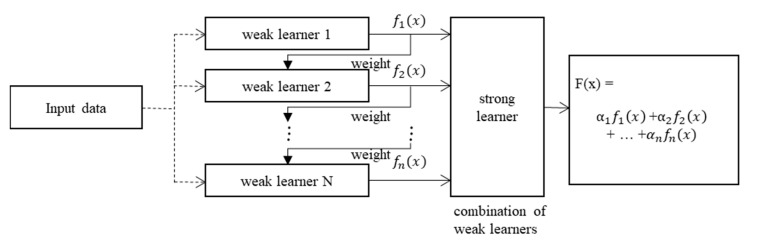
Procedure of an ensemble from an input sample.

In the ensemble method, numerous weak learners are combined to create a learner featuring strong predictive power. In this study, the regression tree method was used as the ensemble learner, and the boosting method was used for combining the models used. The boosting method performs slightly better than the bagging method and exhibits a higher predictive power. In addition, the boosting method can be used to compensate for shortcomings in learning the next regression tree by assigning weights to reduce errors between the prediction and output values of the first regression tree. The least-squares method was used for calculating the error. Least-squares boosting is a sequential ensemble method that sequentially builds a decision tree because it compensates for errors in the previous tree as shown in Figure 6; it is defined as Equation (7) [38,39]:

(7) $$F_{T}\left(x\right)=\overset{T}{\underset{t=1}{\sum }}f_{t}\left(x\right)$$

where *x* is the input variable, $f_{t}$ is the weak learner, and $F_{T}\left(x\right)$ is the strong learner.

Each weak learner produces a hypothesis $h(x_{i})$ as the output for each training set sample. At each iteration step t, one weak learner is selected, and a coefficient $\alpha_{t}$ is assigned to minimize the sum of the training errors of the final t-step acceleration classifier, which is defined as follows:

(8) $$E_{t}=\underset{i}{\sum }E\left[F_{t-1}\left(x_{i}\right)+\alpha_{t}h(x_{i}\right)]$$

where $F_{t-1}\left(x\right)$ is the accelerated learner generated up to the last training stage, E(F) is the error function, and $f_{t}\left(x\right)=\alpha_{t}h\left(x\right)$ is the weak learner currently considered for addition to the final classifier. At each iteration step in the training process, a weight with the same value as the current error $E\left(F_{t-1}\left(x_{i}\right)\right)$ for the training data set is assigned to the next data set, in order to compensate for the errors.

### 3.4. LR

LR of ML is a method of predicting values based on a linear function similar to the LR method [40]. However, because various optimization algorithms and cross-validation are employed, LR can achieve a higher predictive power than normal LR. In LR, the number of input data is indicated as n; each input datum is expressed as $x^{\left(i\right)}=\left[x_{1}{}^{\left(i\right)},x_{2}{}^{\left(i\right)}\right]$ using index *i*, and the label (actual value) is expressed as $y^{\left(i\right)}$. During model training, the error is typically determined using a loss function, such as the expression defined in Equation (9):

(9) $$loss\;function=l^{\left(i\right)}\left(\omega ,b\right)=\frac{1}{2}{\left({\hat{y}}^{\left(i\right)}-y^{\left(i\right)}\right)}^{2}$$

where 1/2 (a constant value) is added to enhance the ease of computation by yielding a value of 1 when the 2D function is differentiated. The smaller the error value, the higher the similarity between the predicted value and the actual value; if the two values are equal, the error will be zero. LR is used for training a model by determining the model parameter $\omega^{*},b^{*}=argminL\left(\omega ,b\right)$, which minimizes the average loss of input data.

## 4. Results and Discussion

### 4.1. Experimental Variability of Static and Dynamic Tests

In this study, the coefficient of variation (COV), which is equal to the standard deviation (i.e., *σ* divided by the average values of the specimen set *μ*), was used to evaluate the experimental variability of the static and dynamic measurements of the concrete specimens. Outliers were confirmed using the modified Z-score method and excluded from the analyses [41]. The statistical parameters—COV and *μ*—of the obtained static and dynamic experimental results are presented in Table 2.

**Figure 7:**
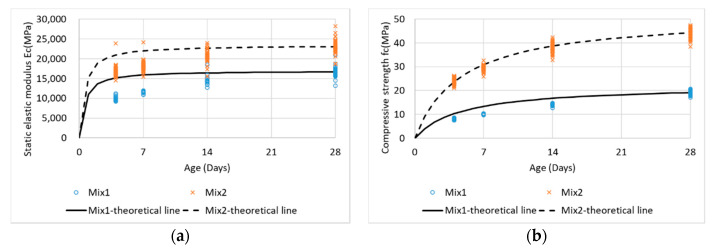
Comparison of experimental and theoretical values for Ec and fc. (**a**) Static elastic modulus; (**b**) Compressive strength.

The COV of *Ec* ranged from 2.99% to 8.50%, and the COV of *fc* ranged from 2.41% to 5.34%. When determining the COV of a slightly higher *Ec*, the opposite side of the test specimen was slightly skewed such that the deformation during compression was nonuniform. The error due to the test appeared to have a more significant effect on the *Ec* results than the *fc* results. In Figure 7 and Table 3, the theoretical (ACI 209R) and experimental values for *Ec* and fc are compared. As presented in Figure 7 and Table 3, concrete with FA and GBFS has characteristics such that the development of the initial elastic modulus and strength develop later than those of normal concrete, but the long-term elastic modulus and strength are increased. In addition, it has the advantage of being suitable for mass concrete because it generates less heat of hydration.

The COV values of *Ed.Vp* determined through the P-waves ranged from 2.17% to 5.99%, and the COV values of *Ed.LT* and *Ed.TR* obtained through the longitudinal and transverse resonance frequency tests were 2.13–4.37% and 2.51–4.69%. The COV values of *Ed.Vp*, *Ed.LT*, and *Ed.TR* were reasonably consistent; however, the COV values of *Ed.Vs* determined through the S-waves ranged from 1.87% to 13.59%, which were higher than those obtained using the other methods. This was because it was often difficult to accurately detect the initial arrival time of the S-waves in early-age concrete, owing to the interference between P-waves and S-waves. Furthermore, even when the S-wave transducer was used, low-amplitude P-waves always appeared with the S-waves in the time domain. This phenomenon was significant owing to the effects of moisture and voids as well as the relatively low compressive strength in the early-age concrete specimens. Therefore, these data sets were not included in the analyses. A thorough effort is required to determine the *Vs* of concrete with relatively low compressive strength.

### 4.2. Relationship between Static and Dynamic Elastic Moduli

Various equations have been proposed for predicting *Ec* using *Ed*. Lydon and Balendran proposed the following empirical Equation (10) [11]:

(10) $$E_{c}=0.83E_{d}\left(\mathrm{MPa}\right)$$

Another empirical Equation (11) was specified in British Standards BS 8110 Part 2 [12]:

(11) $$E_{c}=1.25E_{d}-19,000\left(\mathrm{MPa}\right)$$

It should be noted that Equation (11) is not applicable to concrete containing a lightweight aggregate or more than 500 kg per m^3^ of cement.

Popovics proposed a more general equation for lightweight concrete and normal-weight concrete considering the effect of density [13]:

(12) $$E_{c}=\frac{446.09E_{d}{}^{1.4}}{\omega_{c}}\left(\mathrm{MPa}\right)$$

where $\omega_{c}$ is the density of hardened concrete (kg/m^3^).

**Figure 8:**
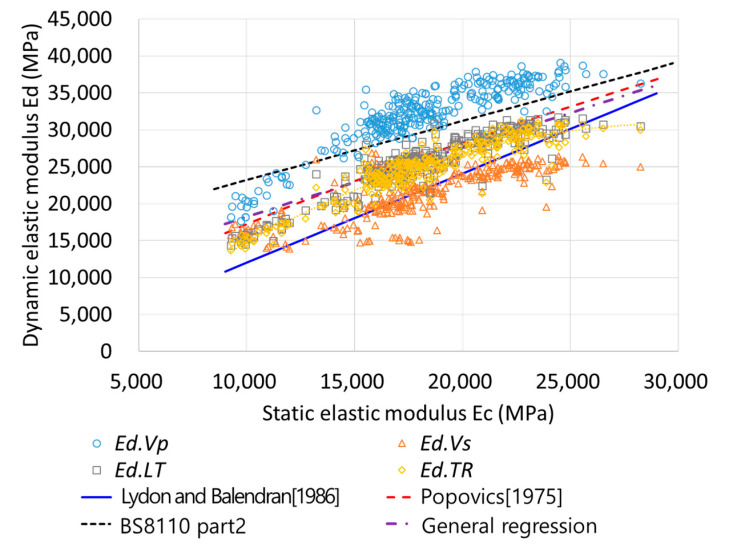
Relationship between *Ec* and *Ed*.

Figure 8 presents the relationship between *Ed* and *Ec* determined using the four methods; based on the figure, it is evident that *Ed* and *Ec* have a nonlinear relationship. In addition, the modulus of elasticity of the concrete, including FA and GBFS, is low in the early-age ranges and at low strength, so it may exhibit nonlinearity. Subsequently, Equations (10)–(12) and the general regression equations were substituted to compare the experimental results with the values obtained using the proposed equations. The equation recommended in BS 8110 Part 2 was located at the top of the data and fits the *Ed.Vp* data; moreover, the Lydon–Balendran equation was located at the bottom of the data and fits the *Ed.Vs* data. The Popovics equation and the general regression equation were centrally located and fit the *Ed.LT* and *Ed.TR* data. As shown in Figure 8, it can be confirmed that the four equations were suitable for predicting *Ed* values; however, as the existing equations were not limited to a specific method, the calculated and measured *Ec* values of the entire set of data were compared for each equation.

The MSE and MAPE were used to verify the errors between the predicted and measured values. The MSE and MAPE are defined by Equations (13) and (14), respectively:

(13) $$MSE=\frac{1}{n}\overset{n}{\underset{i=1}{\sum }}{\left(A_{i}-P_{i}\right)}^{2}$$

(14) $$MAPE=\frac{1}{n}\overset{n}{\underset{i=1}{\sum }}\left|\frac{A_{i}-P_{i}}{A_{i}}\right|$$

where n is the number of experimental data, *Ai* is the measured value, and *Pi* is the predicted value. The MSE, MAPE, and R values for the calculated and measured *Ec* values of the entire data set are listed in Table 4. Among the four equations, the Popovics equation yielded the lowest MAPE value, and the error in its *Ec* prediction was small. For the general regression equation, the MSE value was lower than that for the Popovics equation; however, its MAPE was higher because *Ed* and *Ec* have a nonlinear relationship. Therefore, the results of the general regression equation at a low elastic modulus were higher than those predicted using the Popovics equation. Because of the nonlinear relationship between *Ed* and *Ec*, the general regression equation was considered to generate a larger error than the Popovics equation within the low elastic modulus range. In addition, as the MAPE and R values for *Ed.LT* and *Ed.TR* in the Popovics and general regression equations exhibited a small error range, the prediction error of *Ec* could be minimized when the Popovics equation was applied only to the resonance frequency test.

**Figure 9:**
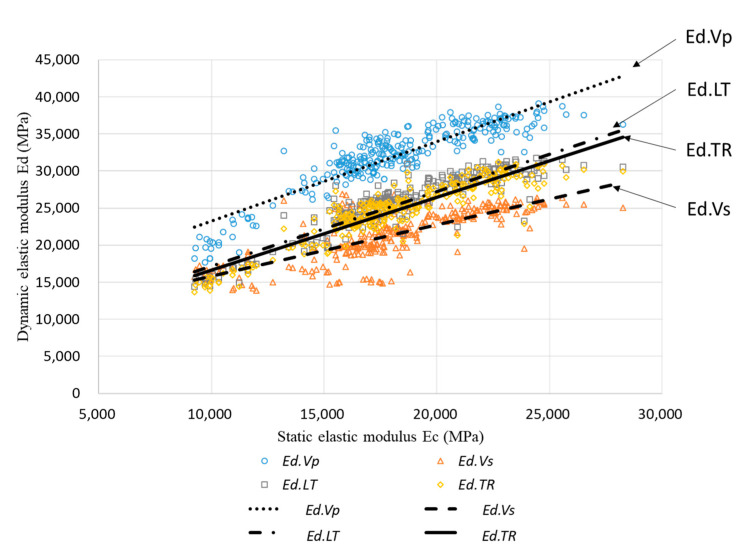
Comparison of individual *Ed* and *Ec* values.

As the existing equations were not applied to a specific *Ed*, the *Ec* value may have a large error value if the equations are incorrectly applied. General regression using individual *Ed* values was analyzed because the *Ed* measured using the four NDE methods possessed different characteristics. Figure 9 presents the overall regression results for each *Ed* value. The MSE, MAPE, and R values between the calculated *Ec* and measured *Ec* are listed in Table 5. The data for *Ed.Vp* and *Ed.Vs* exhibited a significant variance, resulting in MAPE values of 8.59% and 12.95%, respectively. However, the data for *Ed.LT* and *Ed.TR* exhibited a small variance; hence, their MAPE values were reduced to approximately 7%.

### 4.3. Comparison between Static and Prediction Elastic Moduli

Recently, studies have been conducted to improve prediction accuracy and data reliability, using ML methods. In this study, four ML methods were applied by combining the four predicted *Ed* value sets to develop a prediction model that is superior to existing models and the general regression model. SVM, ANN, ensemble, and LR were used as the ML methods, and *Ec* prediction and analyses were performed for 15 different sets of combinations. Table 6 lists the MSE and MAPE values of the predicted and measured *Ec* values for the 15 combinations using ML, where A denotes *Ed.Vp*, B denotes *Ed.Vs*, C denotes *Ed.LT*, and D denotes *Ed.TR*.

For the results of the *Ec* prediction using the single *Ed*, C had the lowest MAPE value, and the prediction error increased in the order of D, A, and B. Depending on the applied ML method, the MAPE values differed by approximately 1–2%; however, the order remained the same. The MAPE values of individual variables predicted using the general regression of Table 4 ranged from 7 to 13%; however, the MAPE values of individual variables predicted using ML ranged from 4.5 to 8%. When the ML techniques were used, the reduction in the prediction errors was 2.5–5% greater than that when using general regression; hence, using ML in *Ec* prediction could result in a more accurate prediction. Based on the analysis of the combinations of two variables, the overall MAPE values were lower than those of single variables, and the BC combination was optimal for predicting *Ec*. The prediction accuracy improved because the longitudinal wave (C) and the transverse wave (B) complemented each other. For the combinations of three *Ed* variables, the ABC combination and the BCD combination exhibited the lowest MSE and MAPE values, respectively, based on the ML methods. The optimal combination varied depending on the applied ML method; however, the BC combination significantly contributed toward *Ec* prediction because BC was included in both optimal combinations. For the 15 combination sets, the accuracy of the ANN and ensemble methods improved as the number of variables increased, and the SVM and linear regression methods had similar prediction accuracies in two or more combination sets. Overall, the ensemble method yielded the highest accuracy for all combination sets, among the four methods.

Although an analysis of Table 5 confirmed that the BC combination contributed significantly toward the predicted *Ec* values, it was difficult to determine in detail the individual variables that contributed toward this prediction. Therefore, the relative importance (RI) using the weight of the ANN was calculated to determine the detailed contribution of each variable involved in the combination [42]:

(15) $$RI_{x}=\overset{m}{\underset{y=1}{\sum }}\omega_{xy}\omega_{yz}$$

In Equation (15), RI is the relative importance of the variable, $\omega_{xy}$ is the weight between the input neuron and the hidden neuron, and $\omega_{yz}$ is the weight between the hidden neuron and output neuron.

Table 7 lists the RI values for 11 combination sets using the ANN method. The order of RI in the combination was C, D, A, and B, which was similar to the order of the MAPE for individual variables using ML and the order of general regression. Based on the RI analysis, the RI value of B had a minimal ratio; however, when B is combined with C, it should be used as a combination rather than a single variable because it improves the accuracy by 0.6%.

**Figure 10:**
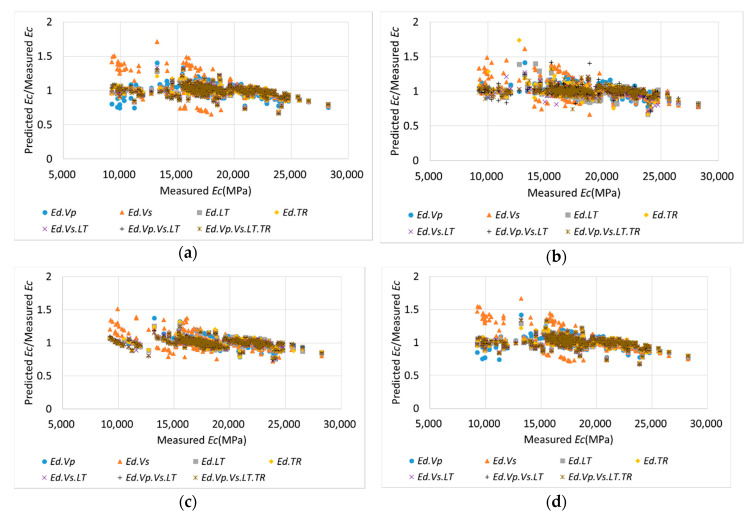
Relationship between measured *Ec* and the ratio of predicted *Ec* to measured *Ec* using ML methods: (**a**) SVM; (**b**) ANN; (**c**) Ensemble; (**d**) LR.

**Figure 11:**
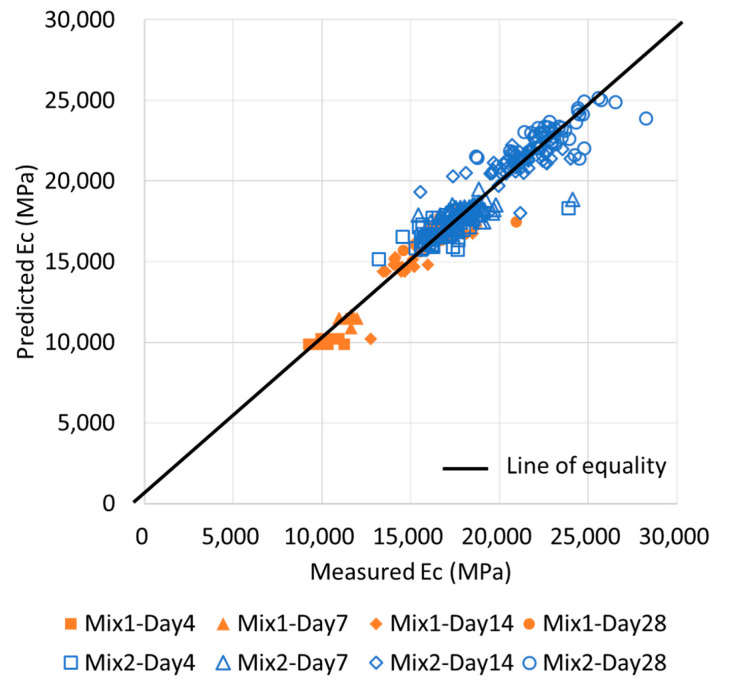
Relationship between measured *Ec* (experimental values) and predicted *Ec* (theoretical values) by the ensemble.

Figure 10 displays the relationship between the measured *Ec* and the ratio of the predicted *Ec* to the measured *Ec* obtained using the four ML methods. The combinations were selected based on the ensemble method, which achieved the highest accuracy. Among all the ML methods, the ratios of the predicted values with two or more variables exhibited smaller variations than those of the single variables. The results generated via the ensemble method were closer to a ratio of 1, compared with those obtained using other methods, and the data distribution was concentrated. Additionally, in the existing linear prediction, it is difficult to consider the error according to age, but the error can be minimized with training by the ML method ANN and the ensemble method. Figure 11 compares the predicted values from the ensemble method with the experimental values for the concrete age. As confirmed from the figure, there is a good correlation, and some data at an early age are out of the line of equality because ultrasonic pulse velocity and resonance frequency methods are suitable for hardened concrete, so it is necessary to develop ML in consideration of concrete age in the future.

Table 8 lists the prediction results for each section of the seven ensemble combinations, where A denotes *Ed.Vp*, B denotes *Ed.Vs*, C denotes *Ed.LT*, and D denotes *Ed.TR*; the section was categorized into three ranges. The differences in error for each section were not large; however, the error was generally high at a low elastic modulus. In particular, the error for *Ec* predicted using B was high at a low modulus of elasticity, because the low elastic modulus data contained numerous early-age data. By contrast, based on the results presented in Table 6, the contribution of *Vs* was large because the accuracy improved when B was included in the combination. Additionally, it was useful for 0.4% when it was 15,000 MPa or more, except for the early-age elastic modulus. Therefore, it could improve the accuracy when performing resonance frequency tests and determining *Vs*, which are the most reliable and widely used methods.

### 4.4. Relationship between Static Elastic Modulus and Compressive Strength

The *Ec* of concrete is determined through static uniaxial compressive tests performed according to ASTM C469/C469M-14 requirements. However, *Ec* is typically determined using compression strength values based on the design codes. Therefore, various equations have been proposed to express the relationship between *Ec* and *fc*. Equation (16), which is proposed in the CEB-FIP and Eurocode 2 codes, is typically applied to normal and high-strength concretes [14]:

(16) $$E_{c}=22,000{\left(f_{c}/10\right)}^{1/3}\left(\mathrm{MPa}\right)$$

Equation (17), which is recommended by the ACI 318 Committee for *fc* values less than 38 MPa, is expressed as follows [15]:

(17) $$E_{c}=0.43f_{c}{}^{0.5}\omega_{c}{}^{1.5}\left(\mathrm{MPa}\right)$$

where $\omega_{c}$ is the density ($1440\leq \omega_{c}\leq 2560\;\mathrm{kg}/m^{3}$).

Equation (18), suggested by the ACI 363 Committee, is used when the value of *fc* lies between 21 MPa and 83 MPa [16]:

(18) $$E_{c}=\left(3320f_{c}{}^{0.5}+6900\right){\left(\omega_{c}/2300\right)}^{1.5}\left(\mathrm{MPa}\right)$$

Equation (19) was proposed by Noguchi et al.; correction factors are applied for ordinary and high-strength concretes based on extensive experimental data [43]:

(19) $$E_{c}=k_{1}k_{2}33,500{\left(f_{c}/60\right)}^{1/3}{\left(\omega_{c}/2400\right)}^{2}\left(\mathrm{MPa}\right)$$

where *k*_1_ and *k*_2_ are the correction factors for the aggregate and SCM, respectively.

**Figure 12:**
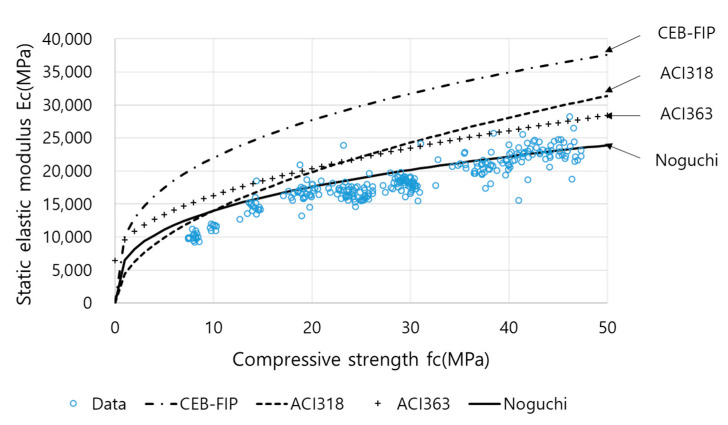
Comparison of the relationships between *Ec* and *fc*.

Figure 12 depicts the relationship between *Ec* and *fc* in accordance with the ASTM C469 and ASTM C39/C39M-14a standards. Equations (16)–(19) were substituted for comparing the predicted values with the measured values. For Equation (19), *k*_1_ = *k*_2_ = 0.95 was employed because materials similar to crushed cobblestone and ground-granulated blast-furnace slag were used.

The MAPEs between the *Ec* calculated using Equations (16)–(18) and the measured *Ec* were 70%, 27%, and 35%, respectively. However, Equation (19) yielded an MAPE of 10%; hence, it was the most suitable equation for predicting *Ec* values. The equation proposed by Noguchi et al. was more suitable than the equations used for ordinary Portland cement, such as CEB-FIP, ACI 318, and ACI 369; this is because the aggregate and SCMs were similar and appropriate correction factors were applied. Therefore, *Ec* and *fc* exhibited a strong correlation, and the values were within a reasonable range.

### 4.5. Relationship between Dynamic Elastic Modulus and Compressive Strength

**Figure 13:**
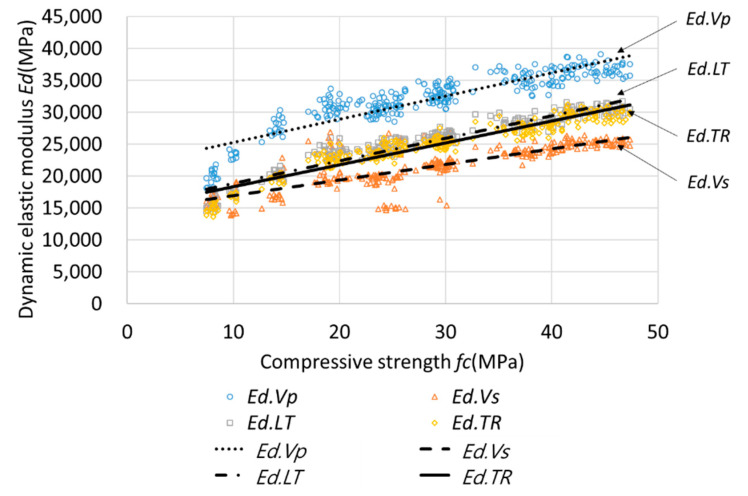
Analysis of general regression for individual *Ed* and *fc*.

Figure 13 presents the relationship between *fc* and *Ed*. The relationship between *Ed* and *fc* was linear when the compressive strength exceeded 20 MPa; however, this relationship was nonlinear at lower strength values, especially due to the concrete mix with FA and GBFS. The *fc* value was calculated using the general regression equations for individual *Ed* values, and the results of the comparison with the measured *fc* values are listed in Table 9. As the *fc* values calculated using all the *Ed* values resulted in large MSE and MAPE values, it was unsuitable to use *Ed* values to compute *fc*. Based on the results of the analysis of individual *Ed* values, the data for A and B exhibited a large variance and MAPE values of 23.54% and 23.64%, respectively. However, the data for C and D exhibited a small variance and MAPE values of approximately 15% each. The MSE difference between A and B was more than twice the A value; however, their MAPE values were almost equal (Table 9). The reason for this difference was that A exhibited a significant variance for all ranges, whereas B exhibited a small variance, except for the early-age ranges.

### 4.6. Comparison between Predicted and Measured Values of Compressive Strength

**Figure 14:**
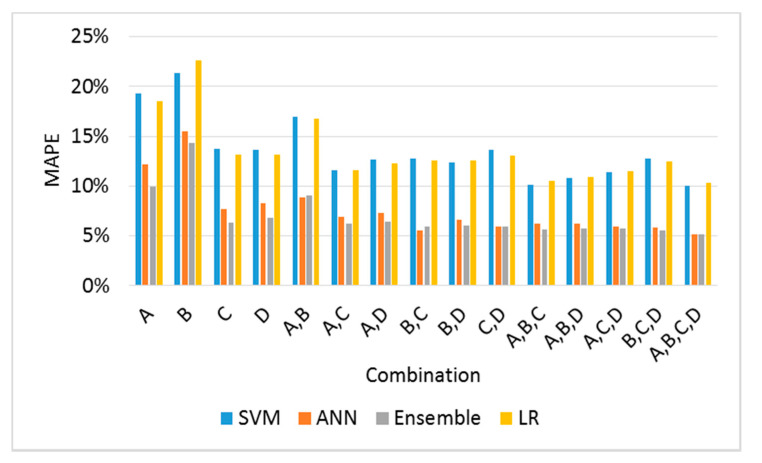
Comparison of MAPE for combinations using four ML methods.

The MSE and MAPE values for the predicted and measured *fc* results for 15 combinations were obtained using four ML methods (Table 10 and Figure 14). For the *fc* prediction results using individual *Ed* values, the MAPE value of C was the lowest, and the prediction error increased in the order of D, A, and B. The MAPE values of the individual *Ed* predicted using the general regression equation presented in Table 9 ranged from 15.3% to 23.5%. However, the MAPE values of the individual *Ed* predicted using ML varied from 6.2% to 22.5%. The SVM and LR methods reduced the MAPE by 2%, as compared to the general regression. However, when the ANN and ensemble methods were used, the MAPE decreased by 8%. Therefore, the ANN and ensemble methods can be used to predict *fc* more accurately. The SVM and LR methods exhibited a significant variation in MAPE for two or more combinations, and the ANN and ensemble methods exhibited decreases in the MAPE as the number of variables increased. For the ANN and ensemble methods, the optimal combinations using two and three variables were BC, ABC, and BCD. Hence, the BC combination contributed significantly to the accurate prediction of *fc*, because all the optimal combinations included BC. This observation was similar to that for the predicted results of *Ec*.

**Figure 15:**
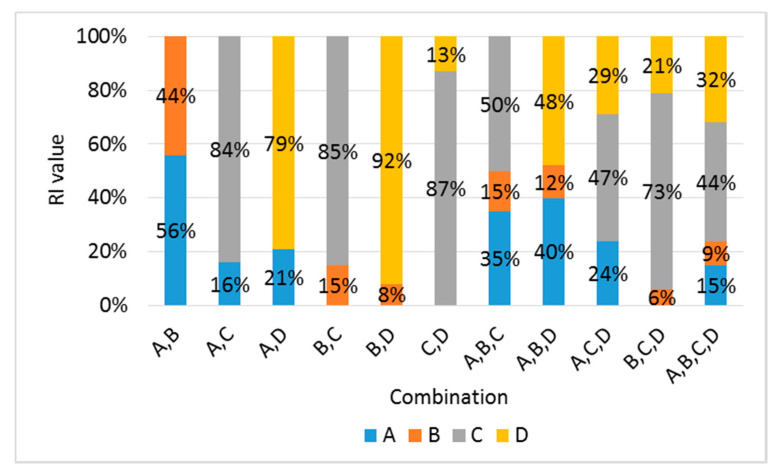
Comparison of RI values using the ANN method.

Table 11 and Figure 15 list the RI values for two or more combinations using Equation (15) derived from the ANN method. The order of RI in the combination sets was C, D, A, and B, which was similar to the trend of the MAPE order for individual variables using ML, the order for the general regression, and the RI order of the *Ec* predicted values.

**Figure 16:**
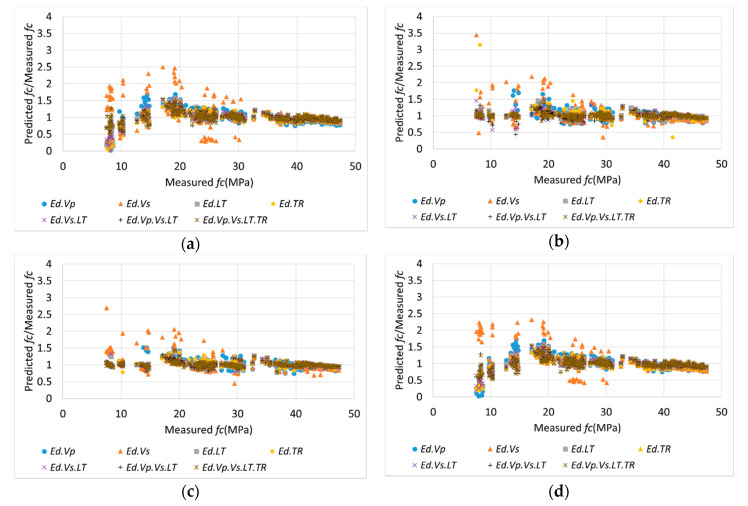
Relationship between the measured *Ec* and the ratio of predicted *fc* to measured *fc* using the four ML methods. (**a**) SVM; (**b**) ANN; (**c**) Ensemble; (**d**) LR.

**Figure 17:**
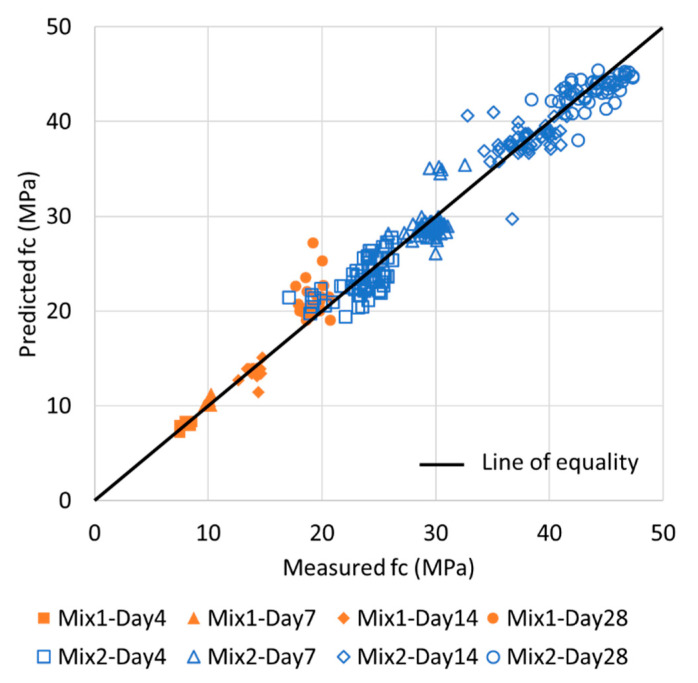
Relationship between measured *fc* (experimental values) and predicted *fc* (theoretical values) by the ensemble method.

Figure 16 depicts the relationship between the measured *fc* and the ratio of the predicted *fc* to the measured *fc* using the four ML methods. The combination sets were selected based on the ensemble method, which achieved the highest accuracy. As B was affected by moisture and voids during the early ages, the MAPE of B was high for the early-age data range; however, at higher strength values (35–50 MPa), B yielded a lower MAPE than A. The results for the ANN and ensemble methods were close to a ratio of 1 for all the ranges, and the variance in the data was small and dense; this resulted in an excellent prediction accuracy. However, the results obtained using the SVM and LR methods had a large dispersion at strengths of 20 MPa and lower. This was because the nonlinearity for the range below 20 MPa was high, as shown in Figure 12 and Figure 13. Furthermore, as the basic functions of SVM and LR are linear, the function might be generalized for the high-strength range, which exhibits linearity and contains numerous data. Therefore, errors in the prediction for low-strength ranges may be high. As a result, the existing linear predictions are difficult for considering the slow development of strength at a low age, but this can be overcome through the use of ML such as ANNs and ensembles and the combination of various *Eds*. On the other hand, the values predicted by ensemble are presented with the experimental results in Figure 17 for the comparison analysis. Similarly, to in Figure 12, it can be seen that the error in early-aged concrete is relatively large; thus, it can be recognized that this approach is more effective for hardened concrete after 14 days of age.

**Figure 18:**
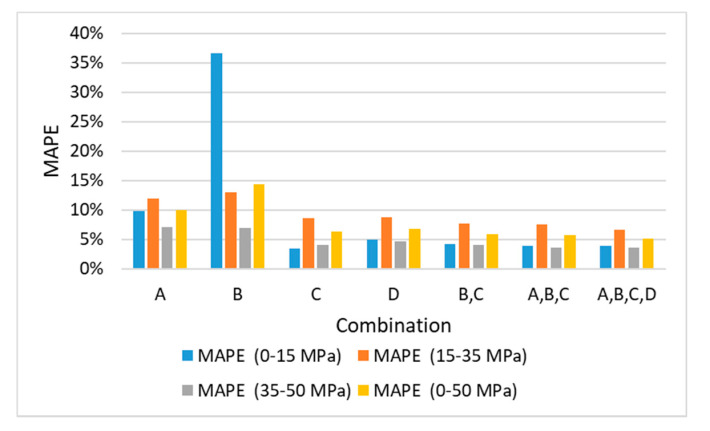
MAPE values for each section of *fc* predicted using the ensemble method.

The MAPE values for various *fc* ranges of the seven combination sets predicted using the ensemble method are presented in Table 12 and Figure 18. The MAPE values for the ranges of 0–15 and 35–50 MPa were similar; however, those for the range of 15–35 MPa increased. For the 15–35 MPa strength range, because the later-age data of Mix 1 and the early-age data of Mix 2 were mixed, the prediction error may be higher, owing to the differences in the mix proportions and curing ages of the data set.

In this study, the correlations between *Ed* and *Ec*, and *Ed* and *fc*, were analyzed using four types of *Ed*. Additionally, four ML methods—SVM, ANN, ensemble, and LR—were applied to analyze the accuracy and RI of each variable combination. The relationship between *Ed*-*Ec* and *Ed*-*fc* was nonlinear in the low-elastic-modulus and low-strength ranges. The *Ec* and *fc* values predicted using the ML methods were more accurate than those obtained using general regression. The ML methods were advantageous over general regression because they allow correction for nonlinearities in the ranges of the early-age strength and elastic modulus. The ensemble method achieved the best performance in predicting *Ec* and *fc*, among the four ML methods. C produced the highest accuracy when used for a single variable; however, it was difficult to predict *Ed* and *fc* as a single variable for B, because the variance in the data was significant, owing to the presence of moisture and voids at the early age. However, B could yield the best accuracy in predicting *Ec* and *fc* when combined with C.

## 5. Conclusions

Currently, it can be difficult or impossible to obtain sufficient specimens by destructive tests according to the ASTM C469 and C39 for the measurement of the Ec and fc required for the design, construction, and maintenance of a structure. Thus, it is necessary to predict them by the non-destructive test (NDT), and one of the most reliable NDT methods is to estimate Ec and fc from the dynamic elastic modulus by ultrasonic pulse velocity or resonance frequency tests. The Ec and fc have usually been predicted from Ed by classical equations or the regression analysis, but these cannot not give an accurate estimation due to the intrinsic effects of each test method and nonlinearity between Ec or fc and Ed. In this regard, this study presented a possibility to improve the estimation of Ec and fc from Ed by a machine learning (ML) algorism based on the results of four types of test on the 282 specimens, comparing with the classical equations such as the Lydon and Balendran equation, Popovics equation, and BS 8110, as well as the regression method. The optimum relationship between Ec or fc and Ed was derived by using the characteristics of Ed from ultrasonic velocity and resonance frequency test methods associated with four ML algorisms. The accuracy of predicting Ec and fc was compared with the general regression and ML based on four reliable Ed measurements, for the complete range of experimental data. The following conclusions have been drawn:

- Among the four Eds, A is the largest, C and D are located in the middle, and B has the smallest value. The correlation with Ec and fc was identified as C > D > A > B.
- The Lydon and Balendran equation yields similar values to the B, and the Popovics equation provides results that are similar to the C and D. In addition, BS 8110 Part 2 yields similar values to the A.
- For the *Ec* and *fc* prediction results, the application of ML improved the accuracy by 2.5–5% and 7–9%, respectively, as compared with general regression.
- When *Ec* was predicted using the four *Ed* values, the order of accuracy of a single variable was C > D > A > B. The combination sets (B, C), (A, B, C), and (A, B, C, D) yielded the highest accuracy. Moreover, only the combination of B and C attained an accuracy level of up to 4.18%. Although B contained large-variance data for the early-age concrete, it complemented the most widely used and reliable C method.
- When *fc* was predicted using the four *Ed* values, the order of accuracy of a single variable was C > D > A > B, which is similar to the order for the *Ec* values. The combinations of (B, C) and (B, D) were optimal, yielding accuracies of up to 5.90%. Although the variance in the B data was large for the prediction of *fc*, B was considered to be useful in supplementing the C and D prediction methods and predicting the resonance frequency test results.
- For predicting the *Ec* and *fc* values, the RI values of B/C were 26%/74% and 15%/85%, respectively. Although B did not contribute significantly, it influenced the improvement in accuracy by 0.6%. When compared with respect to the sections, it was useful for 0.4% when it was 15,000 MPa or more, except for early-age strength.
- With the existing linear prediction, it is difficult to overcome the differences in the characteristics of the eigen data of A, B, C, and D and use them as representative values. However, if ML is used, the representative value of Ed can be calculated through the experimental values of the ultrasonic pulse velocity and resonant frequency test, and it can be very advantageous for predicting *Ec* and *fc*. Throughout this study, C was the best of the four dynamic elastic moduli commonly used, and gave the highest contribution in various combinations. B contributed to predictive accuracy through a combination. The possibility of the use of two or more variables has been verified by ML; if various data on age, temperature, etc. are secured, it will be possible to use a correction for these and to generate more accurate mechanisms and predictive values.
- As shown in Figure 11 and Figure 17, since this study is based on the test methods for the dynamic elastic modulus for hardened concrete, the estimation accuracy tends to be slightly lower for concrete with 4- or 7-day ages. Thus, for a further study, more consideration of other factors such as the type of binder, binder/cement ratio, age, curing temperature, etc. will be beneficial, as including more variables in the ML will help to improve the estimation of *Ec* and *fc*.

## Figures and Tables

**Table 1:** Proportions of the concrete mixture groups ^1^.

ID	Cement Type	W/B	S/A	W	C	S	G	Unit Quantity (kg/m^3^) Mineral Admixture		Chemical Admixture	
								FA	GBFS	AE (Binder%)	SP (Binder%)
Mix 1 (20 MPa)	Type I	0.45	0.46	259	121	777	934	58	69	0.9	-
Mix 2 (40 MPa)		0.35	0.47	308	166	761	886	81	85	-	1

^1^ SCMs: Supplementary cementitious materials, W: water, C: cement, S: sand, G: crushed cobblestone, FA: fly ash, SC: slag cement, AE: air-entraining agent, and SP: superplasticizer.

**Table 2:** Summary of statistical analysis.

Curing Age / Variable		*fc* (MPa)		*Ec* (MPa)		*Ed.Vp* (MPa)		*Ed.Vs* (MPa)		*Ed.LT* (MPa)		*Ed.TR* (MPa)	
		Mix 1	Mix 2	Mix 1	Mix 2	Mix 1	Mix 2	Mix 1	Mix 2	Mix 1	Mix 2	Mix 1	Mix 2
Day 4	*N*	16	54	16	54	16	54	16	54	16	54	16	54
	*μ*	8.05	24.17	10,030	16,791	19,766	30,953	16,273	19,184	15,455	24,310	14,939	23,878
	*COV*	3.86	5.04	5.2	7.63	5.99	4.1	3.96	13.59	4.37	3.69	4.69	3.89
Day 7	*N*	9	55	9	55	9	55	9	55	9	55	9	55
	*μ*	10.05	29.49	11,526	18,190	23,367	32,912	15,664	22,036	17,262	26,129	16,876	25,252
	*COV*	2.41	3.63	2.99	6.57	2.17	3.73	13.17	8.33	2.56	2.31	2.51	2.62
Day 14	*N*	16	50	16	50	16	50	16	50	16	50	16	50
	*μ*	14.08	38.09	14,666	20,874	27,706	35,191	17,710	23,913	20,259	28,542	19,636	27,516
	*COV*	3.69	5.34	8.50	7.02	4.71	3.35	12.38	2.77	3.49	2.47	3.38	2.97
Day 28	*N*	32	50	32	50	32	50	32	50	32	50	32	50
	*μ*	19.19	43.94	16,699	23,085	30,830	36,870	21,019	25,329	23,705	30,375	22,769	29,670
	*COV*	4.35	4.78	7.54	7.12	3.73	2.52	12.58	1.87	3.65	2.13	3.89	2.68

**Table 3:** Comparison of experimental (mean) and theoretical values for Ec and fc.

Type	Static Elastic Modulus				Compressive Strength			
	Day 4	Day 7	Day 14	Day 28	Day 4	Day 7	Day 14	Day 28
Mix 1 (Theoretical)	15,182	15,913	16,429	16,699	10.27	13.37	16.73	19.14
Mix 1 (Experimental)	10,030	11,526	14,666	16,699	8.05	10.05	14.08	19.19
Mix 2 (Theoretical)	20,987	21,998	22,711	23,085	23.75	30.91	38.69	44.25
Mix 2 (Experimental)	16,791	18,190	20,874	23,085	24.17	29.49	38.09	43.94

**Table 4:** Correlation analysis for predicted and measured *Ec* values obtained using the proposed equations and the general regression equation.

Equation Converting *Ed* into *Ec*	MSE	MAPE	R
General regression	2.09 × 10^7^	20.38%	0.6306
Popovics [9]	2.20 × 10^7^	19.22%	0.6019
Lydon and Balendran [7]	2.43 × 10^7^	22.94%	0.6306
BS 8110 Part 2 [8]	5.22 × 10^7^	37.46%	0.6306

**Table 5:** Analysis of correlation between predicted *Ec* and measured *Ec* using general regression for each variable.

Variable	MSE	MAPE	R
*Ed.Vp*	3.37 × 10^6^	8.59%	0.8965
*Ed.Vs*	1.02 × 10^7^	12.95%	0.7585
*Ed.LT*	2.51 × 10^6^	7.06%	0.9198
*Ed.TR*	2.58 × 10^6^	7.07%	0.9178

**Table 6:** *Ec* results for the 15 combinations predicted using four machine learning (ML) methods.

No.	Combination	SVM		ANN		Ensemble		Linear regression	
		MSE	MAPE	MSE	MAPE	MSE	MAPE	MSE	MAPE
1	A	2.75 × 10^6^	7.16%	2.28 × 10^6^	5.65%	1.90 × 10^6^	5.47%	2.74 × 10^6^	7.11%
2	B	6.25 × 10^6^	10.76%	4.46 × 10^6^	8.71%	3.46 × 10^6^	8.06%	5.94 × 10^6^	10.74%
3	C	2.14 × 10^6^	5.55%	2.10 × 10^6^	5.17%	1.42 × 10^6^	4.50%	2.16 × 10^6^	5.66%
4	D	2.20 × 10^6^	5.63%	2.20 × 10^6^	5.26%	1.50 × 10^6^	4.52%	2.20 × 10^6^	5.70%
5	A,B	2.68 × 10^6^	6.83%	2.27 × 10^6^	5.16%	1.70 × 10^6^	5.32%	2.65 × 10^6^	6.84%
6	A,C	2.19 × 10^6^	5.52%	2.00 × 10^6^	4.76%	1.31 × 10^6^	4.26%	2.20 × 10^6^	5.73%
7	A,D	2.22 × 10^6^	5.67%	2.12 × 10^6^	5.51%	1.41 × 10^6^	4.35%	2.21 × 10^6^	5.81%
8	B,C	2.10 × 10^6^	5.46%	1.78 × 10^6^	4.64%	1.26 × 10^6^	4.18%	2.11 × 10^6^	5.52%
9	B,D	2.13 × 10^6^	5.50%	1.95 × 10^6^	4.98%	1.30 × 10^6^	4.26%	2.12 × 10^6^	5.56%
10	C,D	2.14 × 10^6^	5.54%	1.84 × 10^6^	4.92%	1.32 × 10^6^	4.31%	2.12 × 10^6^	5.56%
11	A,B,C	2.14 × 10^6^	5.46%	1.64 × 10^6^	4.48%	1.22 × 10^6^	4.12%	2.15 × 10^6^	5.55%
12	A,B,D	2.15 × 10^6^	5.53%	1.64 × 10^6^	4.61%	1.26 × 10^6^	4.11%	2.15 × 10^6^	5.62%
13	A,C,D	2.19 × 10^6^	5.50%	1.75 × 10^6^	4.89%	1.27 × 10^6^	4.25%	2.19 × 10^6^	5.68%
14	B,C,D	2.08 × 10^6^	5.45%	1.69 × 10^6^	4.37%	1.24 × 10^6^	4.20%	2.09 × 10^6^	5.50%
15	A,B,C,D	2.13 × 10^6^	5.45%	1.45 × 10^6^	4.33%	1.19 × 10^6^	4.03%	2.14 × 10^6^	5.52%

**Table 7:** Comparison of relative importance (RI) values in the ANN.

**A/B**	**A/C**	**A/D**	**B/C**	**B/D**	**C/D**
68%/32%	27%/73%	47%/53%	26%/74%	29%/71%	75%/25%
**A/B/C**	**A/B/D**	**A/C/D**	**B/C/D**	**A/B/C/D**	
17%/12%/71%	35%/9%/56%	29%/38%/33%	21%/41%/38%	11%/4/%/47%/38%	

**Table 8:** MAPE for each section of *Ec* predicted using the ensemble method.

Combination	0–15,000 (MAPE)	15,000–20,000 (MAPE)	20,000–30,000 (MAPE)	0–30,000 (MAPE)
A	5.43%	5.56%	5.33%	5.47%
B	17.20%	7.06%	5.81%	8.06%
C	4.78%	4.43%	4.51%	4.50%
D	4.73%	4.39%	4.64%	4.52%
B,C	5.25%	4.02%	4.00%	4.18%
A,B,C	5.31%	4.00%	3.79%	4.12%
A,B,C,D	4.67%	3.95%	3.89%	4.03%

**Table 9:** Comparison of MSE, MAPE, and R values for predicted *fc* and measured *fc*.

Variable	MSE	MAPE	R
All data	167.65	43.26%	0.6469
*Ed.Vp*	29.86	23.54%	0.8953
*Ed.Vs*	69.94	23.64%	0.7956
*Ed.LT*	12.98	15.24%	0.9501
*Ed.TR*	13.83	15.34%	0.9471

**Table 10:** Predicted *fc* results for 15 combination sets using the four ML methods.

No.	Combination	SVM		ANN		Ensemble		Linear Regression	
		MSE	MAPE	MSE	MAPE	MSE	MAPE	MSE	MAPE
1	A	24.57	19.29%	17.92	12.22%	11.54	9.95%	24.23	18.48%
2	B	49.87	21.32%	29.50	15.47%	21.78	14.31%	44.77	22.59%
3	C	11.94	13.71%	10.75	7.65%	5.06	6.28%	11.84	13.12%
4	D	12.75	13.67%	10.99	8.23%	5.75	6.80%	12.48	13.18%
5	A,B	22.21	16.93%	13.50	8.86%	9.34	9.05%	21.86	16.76%
6	A,C	10.34	11.57%	6.26	6.88%	4.57	6.19%	10.26	11.57%
7	A,D	12.32	12.67%	7.61	7.32%	5.14	6.41%	11.94	12.32%
8	B,C	11.16	12.75%	5.23	5.57%	4.50	5.90%	11.13	12.52%
9	B,D	11.38	12.37%	5.18	6.61%	4.48	6.03%	11.34	12.53%
10	C,D	11.93	13.61%	5.47	5.94%	4.51	5.95%	11.81	13.10%
11	A,B,C	9.00	10.10%	4.94	6.20%	4.04	5.66%	8.92	10.48%
12	A,B,D	10.41	10.85%	5.10	6.23%	4.15	5.71%	10.07	10.88%
13	A,C,D	10.28	11.36%	5.06	5.91%	4.06	5.71%	10.26	11.54%
14	B,C,D	11.14	12.79%	4.77	5.84%	4.00	5.59%	11.02	12.47%
15	A,B,C,D	9.05	10.01%	4.07	5.19%	3.56	5.19%	8.80	10.36%

**Table 11:** Comparison of RI values using the ANN method.

**A/B**	**A/C**	**A/D**	**B/C**	**B/D**	**C/D**
56%/44%	16%/84%	21%/79%	15%/85%	8%/92%	87%/13%
**A/B/C**	**A/B/D**	**A/C/D**	**B/C/D**	**A/B/C/D**	
34%/15%/50%	40%/12%/48%	24%/47%/29%	6%/73%/20%	15%/9/%/44%/32%	

**Table 12:** MAPE values for each section of *fc* predicted using the ensemble method.

Combination	MAPE (0–15 MPa)	MAPE (15–35 MPa)	MAPE (35–50 MPa)	MAPE (0–50 MPa)
A	9.80%	11.95%	7.04%	9.95%
B	36.62%	12.95%	6.89%	14.31%
C	3.44%	8.59%	4.06%	6.28%
D	4.98%	8.78%	4.64%	6.80%
B,C	4.24%	7.66%	3.99%	5.90%
A,B,C	3.98%	7.50%	3.65%	5.66%
A,B,C,D	3.96%	6.62%	3.57%	5.19%
